# *Reports on Cities, Towns, and Districts*.—Huddersfield

**Published:** 1857-07

**Authors:** Samuel Knaggs


### SANITARY AND SOCIAL SCIENCE.

### REPORTS ON CITIES, TOWNS, & DISTRICTS.

### THE SANITARY CONDITION OF HUDDERSFIELD.

By SAMUEL KNAGGS, Esq.

(*Concluded from* p. 79.)

GROSS MORTALITY. The total mortality in Huddersfield, during the five years ending December 1855, was 4,052 : the greatest mortality occurred in 1853, when the deaths amounted to 870 ; the lowest in 1855, when they numbered only 713 ; the average mortality of the five years being 810, or, one in every thirty-eight of the population. The average mortality throughout England of the fourteen years, 1838-51, is at the rate of 2·234 per cent., or, 672 equivalent deaths for the number of the population of Huddersfield ; being in the proportion of 1 to 46. From this it is evident that 138 lives are annually lost in Huddersfield above the average mortality of England. Looking at the density of population in England, we find that each individual bears relation to two acres of surface ; whereas, in Huddersfield, there are eight persons to each acre. This consideration, however, is not sufficient to explain the reason of this high rate of mortality ; because we see from the returns of the Registrar General, that there are many towns in which the density of population is as great as Huddersfield, but where the gross rate of mortality is considerably less. Thus Cheltenham, with nine persons to each acre, loses only one in every 50 ; and Southampton, with thirteen to the acre, one in 41. The mortality of Huddersfield is identical with that of Birmingham and Shoreditch ; yet the former numbers 65, and the latter 161 persons, to each acre ; and in the city of London, with 129 to the acre, the deaths are one in 39, or eighteen deaths fewer than Huddersfield. The mortality in Huddersfield contrasts favourably with that of many of our manufacturing districts ; thus, in the districts of Leeds and Liverpool, the deaths are one in 30, the former numbering forty-eight persons to the acre, the latter 116 ; whilst in the Manchester district, which has eighteen to the acre, the deaths are one in 29, which gives a mortality of 252 in excess of Huddersfield.

But if we contrast the mortality of Huddersfield with that of a tolerably healthy agricultural district, we shall have little cause for congratulation, but much for serious reflection, upon the sources which originate such wholesale sacrifice of human life. In Huntingdon, for example, the deaths are as one to 59 of the living; and in Cranbrook in Kent, one in 61: which would give 304 deaths less than Huddersfield, with an equivalent population.

Mortality of Children. In analysing the mortality tables, we are struck with the large proportion of deaths occurring in early life. Taking the mean of the three years 1853-54-55, 353 children die annually in Huddersfield, under the age of five years. An average of the births in 1851 and 1852, gives a mean of 1,216; so that we may estimate, that out of every seven children born in Huddersfield, two die ere the close of the year. The mortality throughout England, at the same period of life, is as 2 to 8, and in healthy country districts, as about 2 to 10: thus we are enabled to detect one source of the high rate of mortality, inasmuch as we find that Huddersfield, proportionally to its births, numbers 49 deaths in excess of the average of England, and 110 in excess of healthy country districts. In some manufacturing districts the infantile mortality is still higher: one half of all the children in Manchester die before they attain the age of five years.

Mortality of Adults. In comparing the mortality of more advanced life in Huddersfield with that of England, we find between the ages of five years and twenty-five, a slight excess on the side of Huddersfield; 118 being the mean annual loss of this town. A still greater mortality is shown between the ages of twenty-five and forty-five: it being at the rate of 12 per cent. throughout England, but ranging as high as 14 per cent. in Huddersfield. After forty-five years, the excess of deaths is on the side of England; for at all subsequent ages we find them in the proportion of 33 per cent. of the gross mortality, whereas in Huddersfield they number only 25 per cent.

Mortality of Sexes. In the three years, 1,246 males, and 1,121 females, died in Huddersfield. In relation to sex, we see that the greatest number are males, but contrasted with the experience of England, the excess of deaths in females is on the part of Huddersfield.

Analysis of the Classes of Disease. In examining the comparative fatality of the several classes of disease, we remark at once the great mortality from tubercular disease, which in the three years, 1853-54-55, gives an annual mean of 167 deaths, or 23 per cent. of the deaths from all causes. In England it rates at 14, and in the West Riding at 16 per cent., on an average of four years: so that from this class of disease alone, we have a yearly loss of 72 deaths above England's average. The diseases of this group are consumption, mesenteric disease, and scrofula. From consumption alone, 246 persons died in the two years 54-55, of whom 122 were males, and 124 females: of this number, 101 died under the age of twenty-five years, and 195 under forty-five, so that nearly four-fifths of the deaths from this cause were in comparatively early life. Eighty-two deaths occurred in workers in cotton, silk, and woollen, 42 in labourers, and others engaged in out-door employments, 60 in wives and children of labourers and mechanics, and 62 in all other classes. There is no doubt that many of those here classed as the wives and children of mechanics, for want of more correct data, should appear as workers in cotton, woollen, or silk; but I think that in the last group there is sufficient to justify the inference that the high rate of mortality from this disease does not depend upon causes inherent to the locality itself: the group (62) is formed by small shop-keepers principally, and very few of the upper classes are included. It would have been interesting to know the relative mortality from this disease amongst the workers in cotton, woollen, and silk, and to have compared it with the numbers employed in each department; but the occupations are too loosely returned to obtain anything like a trustworthy approximation to the truth. From diseases of the respiratory organs (excluding consumption), Huddersfield loses 101 annually, or 12½ per cent., which is exactly the same as that maintained by an average of England: this, I think, still further supports the view, that the excess from consumption is from other cause than locality. The deaths from zymotic diseases in Huddersfield, during the three years, 1853-54-55, give an annual mean of 172, bearing a relation of 22 per cent. to all deaths: contrasted with the experience of England, during the four years, 1849-52, we find an average of 97,904 deaths, or 25 per cent. From this it would appear that the balance of this important class of disease is in favour of our town, but it must be borne in mind that the comparison is not instituted between the same years, that the years 1854-55 were unusually healthy years throughout the country generally, whereas the four years of England's experience show a heavy mortality, and of course indicate a high rate of zymotic disease. It would, I think, be found, if practicable to institute an exact comparison at a period when zymotic diseases were rife throughout the country, that the experience of Huddersfield would show itself above, rather than below, the average ratio.

Thus the mortality from fevers during these three healthy years, averaged as high as 5¾ per cent., whilst that of England in the four unhealthy years, was only 4½ per cent. The diseases of the zymotic group are in some years virulently epidemic, and when cholera, influenza, fevers, and infantile diseases of this class are so, the rate of mortality rises to an extreme degree, and there is little doubt that the influences which are capable of generating fevers to excess in healthy years, would be quite adequate to raise the rate of mortality in every direction, given a sufficient quantity of the specific virus to set the wheel in motion. Diseases of the nervous system show a relative mortality of 13 per cent. in England, whereas in the three years, '53-55, in Huddersfield, they mount to 18 per cent. Of this group, 85 deaths occur in the year from convulsions, the greater part of which takes place in childhood. Diseases of the urinary organs are more frequent here, bearing the proportion of 1 to 73, whilst in England there is one death from this cause in every 115. Deaths from violence and external causes are also slightly above the average. One mother dies in child-birth to 173 children born alive in Huddersfield; whereas in England, the proportion is as 1 to 192. In this examination of the proximate causes of death, we must not forget what has before been alluded to, that the advantage has always been on the side of Huddersfield, from the necessity which has existed for contrasting the results of an unusually healthy time, with the last report of the Registrar General, which only gives information up to 1852. If, therefore, in this comparison, the rate of special mortality is in excess, we must allow for a still greater augmentation, when the conditions for analysis are more equitably adjusted.

Causes producing Disease. Three hundred and fifty children die every year in this town; many of them from inherited weakness, many from zymotic diseases, but by far the greatest proportion from preventible disease, engendered by improper food and general mismanagement. The period of infancy ought not to be fraught with such extreme peril to the healthy babe; it is exempt from many of those causes which sow the seeds of mortality in the adult, and when children, originally robust, are nurtured sensibly and well, the mortality, except from epidemic disease, is very trifling. It has often fallen to my lot to see fine healthy children at birth swept off in a few days, and sometimes even hours, by convulsions, brought on by the mistaken, but intended, kindness of over-feeding. No sooner is the little creature born, than its solicitous friends, brimful of medical superstitions, and anxiety to do their part in the business, begin to cram it with butter and sugar, bread sops, or gruel, or some similar abomination. Naturally enough, this begets the stomach-ache, to relieve which, each adviser has some favourite panacea, generally a form of opiate—a Dalby, or a Godfrey. If the child is fortunate it gets (as sometimes happens) an overdose, which at once relieves it of its troubles, and the world of its screams; otherwise present relief is purchased by another bill upon the stomach, which soon requires speedy payment: in this way it becomes more and more deranged, the nervous system is involved, and a desperate struggle ensues between the vigorous efforts of nature to repair, and these unintentional, but well directed, powers of art to destroy. With astonishing tenacity of life, some infants fight their way through fields more fatal than those of Marathon, much enfeebled by the severe discipline to which they have been subjected, but every succeeding month, instead of strength brings weakness; experience of the child's sufferings has not awakened the mind to a knowledge of the source from whence they spring—improper feeding began the mischief, the same thing keeps it up, and if life is not, as it were, thrown up in disgust, by some violent convulsion, the child dwindles almost to a skeleton, and sinks from tubercular disease or inanition. This is no highly coloured picture; I am satisfied that more than half of the children that die in Huddersfield, and throughout the country, would live to become men and women, if the nurseries of our country were better managed, and children were treated in accordance with the dictates of reason and common sense. But we have seen that the mortality in Huddersfield in children is above the average, which induces further examination. We consider that some excess may be accounted for by the very imperfect medical attendance which the sick receive. Nearly 100 deaths, registered every year, are uncertified in this town, more than two-thirds of which are children: by uncertified we understand, that these children were either entirely destitute of medical attendance, or else, dependant upon that furnished by unqualified practitioners. To this number we must add those numerous instances of cases certified in which the medical man's aid is sought at the last moment, when life has been almost frittered away by neglect or malpractice, and we shall have some conception of one of the causes which raise the rate of infantile mortality.

Births out of Wedlock. The proportion of births out of wedlock is large; thus 4 to the 100 is that registered in London in 1851, whilst in Huddersfield it is as high as 5 per cent; about 7 per cent. being the average of the whole kingdom. This may have some bearing upon the infantile mortality. Another circumstance, which doubtless has much to do with it, is the pernicious practice, largely in vogue in our manufacturing districts, of mothers leaving their young children, either to the care of some neighbour, or sister, perhaps, but a few years older than the child to be looked after, during the day, whilst the mother pursues her accustomed mill occupation. This is evidently an infraction of those domestic duties, which ought to be paramount in the parent's mind, and which, in a great majority of instances, will assuredly entail its own punishment, in the declining strength and premature death of their neglected offspring.

Causes of Adult Mortality. The influences which operate largely in raising the mortality of our adult population, above the limits of the mortality natural to the country, may be classed under three heads, viz., the general, the social, and those dependent upon occupation. The general influences include atmospheric impurity, unwholesome and adulterated food, badly drained, ill ventilated, and ill lighted dwelling houses. Amongst the social influences, we may notice intemperance, sedentary habits, intermarriage between blood relations, and persons of the scrofulous diathesis ; whilst, as resulting from occupation, we have overwork, injurious to all, but destructive to children ; employments entailing constrained and unnatural postures, as with tailors and shoemakers ; overheated rooms ; or atmospheres impregnated with particles of dust, metal, stone, or other gritty impurity.

The atmosphere, though invisible, is essential to life ; its principal use is to supply us with a medium for breathing ; any gaseous, liquid, or solid particle contained in it, foreign to its natural composition, acts either mechanically upon the air cells of the lungs, with which it comes in contact, or else is absorbed into the blood, and thus distributed over the whole body : in the first case, it irritates and inflames the surface—in the second, it vitiates and poisons the system. The sensible causes, which contaminate the atmosphere of Huddersfield, are evident to the most superficial observer : the air in its purity should be evident neither to the senses of sight nor smell, but here it is otherwise. Around us are long chimneys, continually vomiting forth dense masses of black smoke, which gradually mingle with the pure fluid with which God, in compassion to our necessities, has furnished us, and thus we provide a continual irritant for our respiratory organs, an artificial ebon covering for vegetation, and the sure means for a speedy and effectual disfigurement of one of the most beautiful towns in the West Riding of Yorkshire.

The Town Drainage. With respect to the drainage of Huddersfield we speak guardedly, as we have not yet had the opportunity of examining into its details. It appears that all the drainage of the town is discharged by several outlets into the river; one by King's Mill, another near the Shorefoot, and a third near the gas works: now by this means the river is poisoned and rendered impure, before its waters have passed by the town, which might easily have been prevented, by causing all the sewerage to open into the river *below* the town. The Colne is not a tidal river, and therefore if all the sewerage of the town was conveyed along a main sewer to some distance below the town, and there merged into the stream, we should have comparatively pure water, and less injurious exhalations from it. The Colne rises from the heights of Saddleworth and Holmfirth, and brings to us, no doubt, some considerable dye, and other impurity, collected in its course; but that does not excuse our pouring in the excreta of 30,000 persons immediately under our very noses. The perfection of drainage would be, to divert all the sewerage, dyes, and excrement, into a separate channel; to deodorise, precipitate, and dispose of the solid material for the fertilisation of the earth; and to allow the supernatant purified water to empty itself into the river *below* the town. This appears to be done effectively, and without any offence or detriment to the community, at Glasgow; and surely, if this be so, the example is worthy of universal adoption.

In this town, cesspools, or ash pits, connected with privies, are very numerous. These ought never to be permitted in towns, or where any large number of houses are collected together in a small space: their existence is only necessary when the system of drainage is imperfect or incomplete. When there is drainage near a house, a three-inch pot pipe should be laid from the privy to that drain, and the privy itself should be provided with a "Sanitary Pan Closet," consisting of a simple pan and syphon trap, made of glazed earthenware, and which should be attached to the drain. If it is not practicable to lay on water with a cock to such a pan, it must be kept clean by throwing down all the slops and waste water, or at least a pail of water every day. The occasional practice of carrying drains beneath the cellar of a kitchen of a house is very mischievous, and most provocative of fevers; all drains should be carried round the outside of houses, and in rows of houses, the drains should run into a common drain under the backyard, which should run into the street drain at the end of the row. Surface drains should never be permitted to carry off any water from sinks or waterclosets, which should always go to the underground drain; and all gratings leading to drains should be trapped.

House Ventilation. The general plan of house building is good, and the houses of many of our artisans are fitted up admirably; but a little more attention to ventilation, generally, and the lighting of our cellar kitchens, would be beneficial. Many of these cellar kitchens are abodes which the artisan should shun; if the drainage is not exceedingly good, it is in them that the poison is most potent, because least diluted: in them we see our typhus and scarlet fever cases in the severest forms and largest proportion; into many of them the sun-light can but feebly enter, and light is as essential to robust health, as the air we breathe and the food we live on. A great defect in the cellar kitchens of Huddersfield, is not having a sufficiently large area in front of the windows. In London the area is generally about two yards wide, extending along the whole frontage of the house, so as to give a good supply of air and light; the bottom of the area being below the level of the basement floor. But in Huddersfield, a small area, about half a yard wide, and of the length of the window, is placed outside each window, with a grating at top, so that air and light are both obstructed, and the bottoms of these areas are about on a level with the sill of the window. The products of the combustion, both of respiration and of gas light, are poisonous, and therefore unfitted for inhalation. By such simple means, as the fireplace open to the ceiling, Arnott's valve, the syphon tube, or the diaphragm chimney, it is practicable to provide effectually against this source of contamination of the air we breathe; and yet how rarely is it that we find more provision made for the entrance of pure air, and the exit of foul, than is to be found in the bore of the key-hole, the chink of a window sash, or the accident of a fire place.

Atmospheric Self-Purification. There is one point in connection with the purification of the atmosphere, which ought not to be overlooked. The poison of animals is the food of plants—what plants exhale (oxygen) is the respiratory food of man. If, therefore, in our crowded towns we cannot give to each little family a separate garden plot, at least, let us make use of all our open available spaces,—planting them with trees and hardy shrubs, so that, by means of these atmospheric scavengers, the air may be preserved in its purity.

Intemperance. Habits of intemperance are largely prevalent amongst us; and it is to these that we owe our excessive mortality from renal, liver, brain, and perhaps, tubercular diseases. The causes promoting these habits, and through them such wide spread misery, are very numerous. Amongst the most influential, we recognize the great multiplication of beer houses, which cannot be supported by the *legitimate* sale of beer; the meetings of clubs, and friendly societies, and payment of monies in such places; unhappy and badly regulated homes, which drive the man from the fireside circle, to take refuge in the tavern; these are too prevalent in our manufacturing neighbourhoods, and are in great degree traceable to the employment of our young females in factories, which seems a bad preparation for the quiet routine of domestic life: and lastly, the want of provident habits, which the vicissitudes of trade and health require, and the absence of taste for those places which offer innocent and healthful recreation both for mind and body.

So long as intoxicating liquors are procurable, we must expect to have some degree of drunkenness; but if we can divert some of the great influences the vice will considerably diminish, and the disease resulting therefrom. To this end let us encourage those well formed provident societies, which protect the working man against the misfortunes of scanty work and bad health, keep him free from debt and care, and which do not expose him to temptation, by holding meetings at beer houses: let us educate his mind and instincts when young to higher aspirations and nobler tastes, by our day and Sunday schools, mechanics' institutions, concerts, lectures, and free libraries: let us give him bathing places, gymnasia, and parks for his health, happy and healthful homes for his family, and we strike directly at the root of the most fearful social evil, which decimates the lives, while it desolates the happiness of thousands upon thousands of our countrymen.

Overwork. The evils resulting from overwork are to be found in all classes of society: to maintain the body in health, properly apportioned exercise, recreation, and rest of its corporeal and mental functions, are essential. If we neglect these conditions, so necessary to its well being, we introduce an element of decay; and it matters little whether it proceed from the apathy and inactivity of indolence, in its mental or physical agency, or from the opposite extreme of over-excitement and over-taxed activity.

In our crowded seats of manufacture, however, the danger is not so much from the too little, as from the excessive, strain to which both mind and body are subjected: here every faculty is, as it were, lashed into violent exertion, to outstrip in the race of competition. To the necessity for restraining this overwork, which consigned so many, especially amongst young people, to an early grave, we must attribute the interference of the legislature, in the shape of "the Ten Hours Bill." The great points introduced into the factory practice by this measure are, that no children are admitted to factory work under eight years of age; children between the ages of eight and thirteen work from six to seven hours a day, and are besides compelled to have three hours schooling; or they may work ten hours on alternate days, by giving notice to the inspector of the district, in which case they are required to have five hours schooling on the previous day. Females of all ages, from thirteen upwards, and males between the ages of thirteen and eighteen, are called young persons, and work ten and a half-hours a day, for five days, and seven and a half on the Saturday. Males over the age of eighteen are not restricted as to time, and frequently subject themselves to overtime. In an able pamphlet, by Mr. Baker of Leeds, on the "Factory Acts," he tells us that the "duration of life amongst the factory-workers is very nearly the lowest amongst English work-people;" and if this be so, it is clear that much yet remains to be done for the physical protection of our factory workers.

Perhaps of all compelled causes, too prolonged work, either of mind or body, is the most productive of disease; it sows the seeds of decay alike in the peer of the realm, as in the needlewomen, and workers in our factories. For those individuals, who voluntarily impose upon themselves more labour than their strength enables them to support, we can here afford but little sympathy; but we think it is eminently due in those cases, where thousands of our humbler brethren are compelled to earn their daily bread under a fixed system, which necessitates a wear and tear of mind and body, to which no human beings can be subjected without receiving permanent and certain injury. During my few years residence in this town, some improvement has resulted from the operations of a society having a branch here, and called the "Early Closing Association," established for the purpose of shortening the hours of labour throughout the land, by the agency of combined agitation, and the influence of well directed and enlightened public opinion.

Slight reflection assures us how great masses of disease originate directly from ignorance of the most simple and elementary physiological laws of nature; truths which ought to be indelibly impressed upon the mind of every child, but which, in this age of progress, are not thought of sufficient importance to enter into the plan of general education. The result is that even amongst our great ones, upon whose attainments and judgment in other matters we rely with reverence and pride, we find too often, in matters connected with hygiene and medicine, the most lamentable inconsistencies in conduct, and the most degrading superstitions in doctrine. All classes of society suffer from this incomprehensible and serious neglect, of that which should constitute one of the first and leading features of healthy practical education ; but in an especial degree is this so with the poorer classes. A vast proportion of the diseases which beset humanity, would, I am convinced, be effectually put a stop to, if the principles upon which our health depends were well engrafted in the minds of society. I do not think it exaggeration to say, that hundreds of lives are yearly lost in this town, from want of a proper knowledge of the functions and requirements of the human body.

We have at the present time no national plan of education ; therefore it is evident that any steps to remedy this deficiency must be from voluntary effort. Something might be done to encourage a taste for this science, by lectures and popular works on the subject. In the hands of the more advanced student, such text-books as those of Carpenter, Lardner, and others, would furnish ample materials of interest and instruction; but amongst the poorer classes I would suggest the circulation of popular and sound physiological tracts, written with reference to the great questions of child management; laws affecting digestion, respiration, and the nervous system ; drainage, ventilation, and others of the same class, as the means best calculated to convey sanitary instruction to the homes of our humbler neighbours.

The Society of Arts, in the examinations, which it has lately instituted, of candidates from mechanics' institutions, has selected physiology as one of the subjects on which it examines : this is, indeed, a step in the right direction, and I trust it will not be long before physiology will assume the position it ought to occupy, from its importance, in every public and private school throughout the land.

But there are some causes which prejudice the comfort and well being of whole neighbourhoods of individuals, not kept up from want of knowledge that the consequences are disagreeable and injurious to others, nor because the nuisances cannot effectually be checked, but simply because the expense of doing away with the nuisance, is more than the individual creating it is willing or able to encounter.

Now this is evidently a state of things requiring the interference of Government, to compel uniformity of practice throughout the country. It is manifestly unjust that thousands of human beings should be forced to breathe an atmosphere, loaded with unburnt charcoal, or poisoned by the fumes of rivers, rendered pestiferous by the refuse thrown into them, for the convenience of a few privileged individuals : whilst at the same time it is also unfair to expect that steps should be taken in one neighbourhood *alone* to remedy such evils, because the manufacturers would be subjected to a considerable outlay; and if the requirements throughout the country were not alike, those laid under such restrictions, would be placed at a disadvantage in the necessary competition of prices. By the appliance of well tested machinery, it is without doubt that the smoke nuisance can be entirely abated; and experiment has also shown, that is quite practicable to filter the refuse of our dye-houses, and to substitute for it a clear and harmless water, which would admit of being discharged into the river without detriment. Surely, then, it would be within the legitimate influence of Parliament to put its veto upon such causes of atmospheric and river impurity; and if the edict of compulsion is general, the effect upon trade cannot be injurious, whilst to the public welfare it must be greatly beneficial.

ADULTERATION. Another very widely spread evil, which seems to call imperatively for some legislative interference, is the adulteration of our food. This is a great crime, and unfortunately, as unimpeachable evidence has proved, one of very common occurrence. It falls with greater severity upon the poorer classes, and is, undoubtedly, in many of its forms very detrimental to the public health. Perhaps the recommendation to appoint experienced public analysts, to subdistricts throughout the country, to whom suspicious articles of food or medicine might be referred for examination, with the punishment of publicity being given to the names of parties vending the adulterated articles, would be a measure as efficient and satisfactory as could be devised for this grievance.

The proportionate mortality accruing from special occupations, requires more consideration and facilities for investigation than could be compassed in the present sketch; and, indeed, the statistical standards for comparison are not arranged with sufficient exactness in the last census report, to allow any safe practical deduction as to the comparative healthiness of the different departments of our local manufacture. From Thackrah's previous observations, we should infer that the employments in the woollen trade are by no means unhealthful: but overwork, employment in overheated and badly ventilated rooms, and dusty and gritty atmospheres, will add very materially to the rate of mortality.

The continuance of all these causes which influence largely our annual mortality, and yet are easily remediable, is a strong argument in favour of the general appointment of health officers. In London and the surrounding districts, competent persons in each district are appointed, to investigate continually the causes which engender local disease, and to suggest such remedial measures as are likely to check it. The population of our parish is far greater than most of those districts to which officers have been appointed, and, therefore, if human life is as valuable in Huddersfield as in London, their efforts are certainly as much required.

Huddersfield, as we have seen, though very superior to many manufacturing towns, is yet considerably above the mortality of healthy towns ; its deaths from fever cases in a healthy year, are beyond the average proportion throughout England in unhealthy years. It is, of course, impossible to tell the number of cases of fevers, and other zymotic diseases, which actually occur, from the number of deaths registered ; but we must suppose, from the fact stated, that the existence of a vast amount of preventible disease is manifest. Now an officer of health would be acquainted day by day, and week by week, with the then prevailing type of disease, from the weekly registers of deaths, and from the information he would be enabled to organize from the parochial medical officers, and others of his professional brethren. The reason of the preponderance of disease in certain localities would soon be made apparent, and the arrest of a pestilence at the outset, would be the saving of many scores of lives. The duty of a medical officer of health would be to exercise a general supervision over all influences, measures or proceedings, involving the public health, and to furnish periodical reports to the governing bodies of his district. Such a person would have every inducement to bring to light all influences injurious to health and susceptible of improvement ; and besides being qualified by previous training for the task of investigation, he would feel, from a sense of duty, directly interested in their exposure.

Under our present system it is the business of no one to make these inquiries. Should a looker-on have the temerity to assert that the rate of mortality is too high to show a healthy town, and to hint at the existence of imperfections to our governing bodies, his statements are, in nine cases out of ten unless emanating from influential sources, received with ridicule, or treated with contempt.

To the preconceived notions of those, whose minds are constantly engaged upon the improvement and progress of the town, it must be in all its details a model town. Huddersfield ought to be, and I think might be made, a model town, for it has very great natural advantages ; but it will not deserve the name of "model", so long as it continues to lose, annually, one in every thirty-eight of its inhabitants. A constant and independent monitor is wanted to bring this important question prominently forward, and to display it in all its aspects, and till this is done no radical improvement is probable. On this question, peculiarly its own, the medical profession now finds, without doubt, all its pecuniary interest arrayed against sanitary reform.

---

### MISCELLANEA.

